# Oral Clinical and Radiological Signs of Excessive Occlusal Forces in Bruxism

**DOI:** 10.3390/diagnostics15060702

**Published:** 2025-03-12

**Authors:** Adrian Marcel Popescu, Mihaela Ionescu, Sanda Mihaela Popescu, Alin Gabriel Ionescu, Diana Elena Vlăduțu, Monica Mihaela Iacov-Crăițoiu, Alexandru Ștefârță, Luana Corina Lascu, Veronica Mercuț

**Affiliations:** 1 Department of Fixed Prosthodontics, University of Medicine and Pharmacy of Craiova, 200349 Craiova, Romania; smpopescu@mail.com; 2 Department of Medical Informatics and Biostatistics, University of Medicine and Pharmacy of Craiova, 200349 Craiova, Romania; 3 Department of Oral Rehabilitation, University of Medicine and Pharmacy of Craiova, 200349 Craiova, Romania; 4 Department of History of Medicine, University of Medicine and Pharmacy of Craiova, 200349 Craiova, Romania; 5 Department of Dental Prosthetics, University of Medicine and Pharmacy of Craiova, 200349 Craiova, Romania; 6 Department of Dental Technology, University of Medicine and Pharmacy of Craiova, 200349 Craiova, Romania; 7 Department of Radiology, University of Medicine and Pharmacy of Craiova, 200349 Craiova, Romania

**Keywords:** bruxism, excessive occlusal forces, attrition tooth wear, fatigue tooth fracture, abfraction, bone apposition, masseter muscle hypertrophy

## Abstract

**Background/Objectives**: Excessive occlusal forces manifest in bruxism and have consequences on teeth and jaws. The aim of this study was to determine the association of bruxism with clinical and radiological signs of excessive occlusal forces, such as tooth wear, fatigue dental fissures and fractures, abfraction, masseter muscle hypertrophy, and bone apposition at the mandibular angle. **Methods**: This cross-sectional clinical study included 181 patients presented for treatment in a general dentistry clinic. For each patient, data were extracted from the dental chart, as follows: demographic data (sex, age, and smoking), clinical data (number of teeth present, Eichner edentulous score, TWI wear score, number of fractured teeth, number of teeth with abfraction, presence of masseter hypertrophy, presence of hypersensitivity), and radiological data (bone apposition at the mandibular angle). The patients were divided into two groups according to the presence or absence of bruxism. A binomial logistic regression model was run to determine the association between bruxism and clinical and radiological signs of excessive occlusal forces. The data were statistically processed in SPSS. **Results**: In total, 99 women and 82 men with mean age 44.87 ± 12.67 were included in the study. Compared to the group without bruxism, the group of patients with bruxism (39.78%) showed statistically significant higher tooth wear index (TWI) (*p* < 0.0005), a higher number of fractured teeth (*p* = 0.037), a higher number of teeth with abfraction lesions (*p* = 0.001), and a significantly higher bone apposition score (*p* < 0.0005). The binomial logistic regression model showed a high prediction bruxism score for masseter muscle hypertrophy (15 times, *p* < 0.0005), for tooth wear index (almost 7 times, *p* = 0.010), and for bone apposition score (almost 3 times, *p* = 0.044). **Conclusions:** Patients with bruxism showed masseter muscle hypertrophy, higher attrition-type tooth wear index, and more teeth with fatigue fractures and abfractions than those without bruxism. Bruxism clinical signs were positively correlated with a higher bone apposition score.

## 1. Introduction

Bruxism, a masticatory muscle activity that implies jaw movements with hard dental contacts in clenching and grinding [1,2], is a common cause of tooth wear and fatigue tooth fissures and fractures [3,4]. In 2018, an international consensus [2] established the bruxism classification of bruxism as sleep bruxism and awake bruxism, according to an asleep or awake state of mind. Sleep bruxism (bruxism occurring in sleep) is considered a rhythmic (phasic) or non-rhythmic (tonic) masticatory muscle activity [2]. Awake bruxism is considered a masticatory muscle activity with repetitive or sustained tooth contact and/or bracing or thrusting of the mandible [2]. Either way, in bruxism masticatory muscle activity and occlusal forces are higher than physiological activities [5,6]. In both types of bruxism, it is considered that in healthy subjects, bruxism is not a movement disorder, and for sleep bruxism, that is not a sleep disorder [2]. Etiological theories of bruxism include peripheral factors (such as occlusal imbalance) [7], central factors (involving neurotransmitters and the basal ganglia) [8], as well as psychosocial factors (stress, anxiety) [9,10,11,12].

Excessive occlusal forces are defined as occlusal forces that exceed the reparative capacity of the periodontium, resulting in occlusal trauma and/or tooth wear [13]. A controversy exists that bruxism produces excessive occlusal forces. According to Nishigawa et al. [14], the maximum force amplitude detected in bruxism events was much higher than the maximum voluntary bite force during the day, having a value of 111.6% of it in one of the subjects tested, and in others, it was smaller. The data of this study showed that the bite force during sleep bruxism episodes could exceed the maximum bite force during the day, having a destructive potential for teeth, periodontium, and dental restorations.

On the contrary, Calderon et al. [15] compared the maximum bite force measured with a gnathodynamometer in women and men with/without bruxism. They found that the force was higher in men, and between those with and without bruxism, there was no statistically significant difference. Calderon’s study is irrelevant regarding the dental effects of bruxism, because it does not say anything about the muscle activity in the subjects included in the study, so a comparison is to be made between bruxists and those without bruxism in terms of intensity and duration of masticatory muscle activity.

Gender, age, and edentulism are factors that could influence bite force [16]. A series of studies showed that bite force is higher in men than in women [15,17,18]. Age does not significantly influence bite force in individuals with dentitions maintaining at least 20 teeth [19]. The number of missing teeth is negatively correlated with bite force, and bite force is lower, especially when molars are absent [20]. In an MRI study of 747 subjects aged 30–89 years, it was observed that edentulism (reduction in the number of teeth), especially in the molar area, determines the decrease in the muscle mass of the masseter and, consequently, the reduction in bite force [21]. Subjects with Eichner B3 edentulism (19 remaining teeth) had a statistically significantly smaller masseter muscle area than those with Eichner class A2 or one tooth edentulism (27 remaining teeth) [21].

Although controversial, excessive occlusal forces do appear in bruxism. In a comparative study on bite force, the authors showed that in bruxism, maximal bite force was higher because of a significant increase in the occlusal contact area (maximal bite force was computed by multiplying maximal bite pressure and occlusal contact area values) [6]. When excessive occlusal forces work for a long time, they have consequences on periodontal ligaments and create occlusal trauma [13], as well as on teeth enamel and dentine, creating tooth wear (attrition, abfraction) [22,23,24] and fatigue fractures [25,26]. Excessive occlusal forces or occlusal trauma were debated by Fan and Caton in 2018 [13]. Still, they presented only the effects of excessive occlusal forces on the periodontal attachment apparatus (fremitus, mobility, widened PDL space), even though they recognized that there were many other clinical and radiographic indicators of occlusal trauma: occlusal discrepancies, wear facets, tooth migration, fractured tooth, thermal sensitivity, discomfort/pain on chewing, root resorption, cemental tear [13].

According to Bronkhorst et al., the correlation between bruxism and tooth wear is controversial [27]. On the other hand, Chan et al. [28] introduces bruxism into the category of risk factors for tooth wear, as do Oudkerk et al. [29] and Hattab et al. [30]. Others mentioned that the association between bruxism and wear was not well-founded [31]. However, a series of recent studies show an association between bruxism and tooth wear [32,33]. In a video-polysomnographic study, subjects with sleep bruxism and attrition exhibited significantly longer phasic burst durations than those with bruxism and without attrition or controls [33].

Wear facets, abfraction, and fatigue tooth fracture reflect the effects of excessive occlusal forces on hard dental tissue. Clinical aspects of tooth wear in these situations include attrition (occlusal and incisal tooth wear because of direct teeth-to-teeth contact) [34,35] and abfraction (non-carious cervical lesion that appears because of a tooth flexion and enamel and dentin lost from enamel cement junction) [36,37,38]. Multiple mechanisms are implicated in tooth wear in general [39], but erosive tooth wear was considered the most common reason for tooth wear [40]. As tooth’s wear origin is multifactorial, combined mechanisms are involved in tooth surface non carious lesions: stress, friction, and acid wear [41,42]. Fatigue fractures and tooth cracks [43,44], accompanied by tooth crack syndrome with hypersensitivity in biting [45], are also effects of the excessive occlusal forces on the tooth hard tissue [13,46].

On digital panoramic radiographs, areas of bone apposition produced by a high force of masseter contraction because of bruxism can be observed [47,48]. Thus, these authors argue that bone apposition diagnosed radiologically can serve as a diagnostic indicator of bruxism. Since these morphological changes require a long time to occur, they provide data not only on the presence of bruxism but also on its duration.

The objective of the study was to compare the association of demographic, clinical (tooth wear, tooth fractures), and radiological aspects (bone apposition) in two groups of patients with and without bruxism. This comparison could provide important clues regarding the incidence of bruxism in the adult Romanian population, clinical aspects of tooth wear and fatigue tooth fractures in bruxism, concomitantly with the evaluation of the effects of excessive masseter muscle forces expressed by bone apposition at the level of the mandible.

The null hypothesis is that there are no statistically significant differences between patients with bruxism and those without bruxism in terms of the incidence of tooth wear, fatigue tooth fractures, and mandibular bone apposition. Such results would mean that, in bruxism, excessive occlusal forces determined by an intense activity of the masseter masticatory muscles are not exerted.

## 2. Materials and Methods

### 2.1. Study Design, Sample Selection, and Ethical Aspects

The cross-sectional clinical study was conducted between October 2022 and January 2024. Participants included were recruited from adult patients presented for diagnosis and oral rehabilitation treatment in a general dentistry office in Craiova, Oltenia, Romania. This region is located in the south–west part of Romania. Craiova is the county city municipality with auto and electric industries and services. This study was approved by the Ethics Committee of the University of Medicine and Pharmacy of Craiova (no 156/25 July 2022). All patients included in the study previously signed an informed-consent form for treatment and to let their data be used in dental research. Declaration from Helsinki was respected in all stages of the study. Data taken from patient records included the results of the anamnesis, clinical examination, and digital orthopantomography examination, as well as data from the photographic exam. Two groups of patients were included: with and without bruxism.

#### Inclusion/Exclusion Criteria

Inclusion criteria: adult patients aged 20–75 years who presented for dental diagnosis and oral rehabilitation treatment, with ASA status I or II, complete dentate patients, and patients with partial edentulism treated or not.

Exclusion criteria: patients with gross malocclusion (as anterior open bite, unilateral cross-bite, overjet higher than 6 mm), with neurological and psychiatric diseases, with decompensated systemic diseases (over ASAIII), with frequent acid consumption and with gastroesophageal reflux disease/reflux esophagitis, patients with temporomandibular dysfunction, patients with complex oral rehabilitation that involves crowns over all teeth, orthodontic treatments.

### 2.2. Bruxism Diagnostic

A specialist dentist in general dentistry with more than 30 years of dentistry and knowledge in bruxism (SMP) examined all patients and extracted data. The diagnosis of bruxism was established following the anamnesis and clinical examination, without differentiating between sleep and awake bruxism (for that being necessary more tests and a considerable amount of clinical time). The sound noise of teeth grinding or teeth clenching heard by the bed partner was considered an important anamnestic criterion for sleep bruxism. For awake bruxism, reported habitual clenching observed by the patients was considered a significant anamnestic criterion. Clinical signs indicating the influence of mechanical factors in bruxism and listed in STAB (Systematic Tool for Assessment of Bruxism) [49] included tooth wear with attrition characteristics (enamel and dentin wear at the same rate, the presence of matching wear on occluding surfaces, corresponding features at the antagonistic teeth, shiny facets, flat and glossy); the presence of oral mucosa signs as impressions in cheek, tongue, and/or lip; presence of cracks within the enamel; the presence of fracture of cusps or restorations; and the presence of buccal/cervical lesions in premolars and cuspids (abfractions). STAB is a proposed tool for diagnosing bruxism, which includes questionnaire assessment, clinical examination, and instrumental examination [49].

### 2.3. Variables

The variables studied included demographic data (potential covariates—such as age, gender, smoking); clinical data such as the presence or absence of bruxism (dependent variable); as well as independent variables like the presence/absence of masseter hypertrophy, the number of present teeth, the TWI tooth wear index, the number of fractured teeth, the number of teeth with abfraction lesions, the presence/absence of dental hypersensitivity, the Eichner type of edentulism, and the degree of bone apposition at the mandibular angle on orthopantomography. Criteria for bruxism diagnosis included data taken from anamnesis and from clinical exam [2]. Criteria taken from anamnesis were sounds heard by the bed partner for sleep bruxism and the presence of clenching moments during the awake time for awake bruxism, history of cracked teeth, and history of morning pain in the masseter or temporal muscles. Criteria from the clinical exam were masseter muscle hypertrophy, oral mucosa signs of bruxism (linea alba, teeth imprints on tongue or cheeks mucosa), presence of attrition, abfraction, and fissures or fractured teeth.

**Figure 1:**
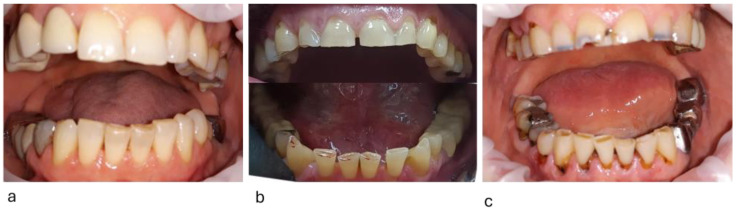
Attrition tooth wear score: (**a**) = low; (**b**) = moderate; (**c**) = severe.

**Figure 2:**
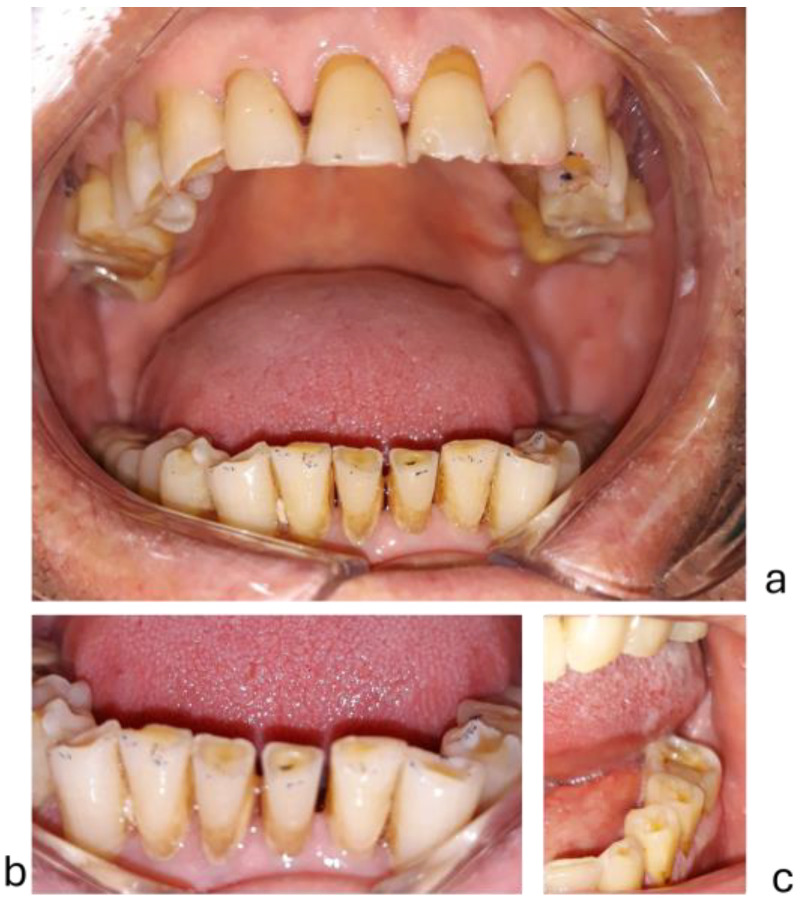
Severe tooth wear in a man: (**a**) = severe tooth wear; (**b**) = lower anterior teeth; (**c**) = lower posterior teeth.

The severity of tooth wear was assessed with the TWI (tooth wear index), an occlusal (palatal) and incisal wear index [50,51]. The scoring method was 0 = no enamel loss, 1 = enamel loss, 2 = dentin exposure on less than 1/3 of the occlusal surface or dentin exposure on the incisal edge, 3 = dentin exposure on more than 1/3 of the occlusal surface or severe dentin exposure without pulp chamber opening, 4 = complete dentin exposure or pulp chamber opening or secondary dentin in the occlusal/incisal area. TWI is a 5-point index from 0 to 4, expressing the severity of tooth wear. An examiner (SMP) recorded TWI scores on the occlusal/incisal surfaces of all teeth, except those with dental or prosthetic restorations. Absent wear = 0, slight wear ≤ 1, moderate wear ≤ 2, severe wear ≤ 3, extreme wear ≤ 4. Clinical aspects of attrition tooth wear are presented in Figure 1 and Figure 2.

**Figure 3:**
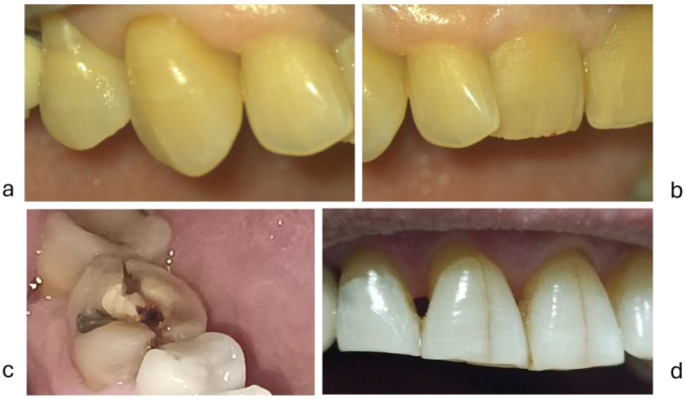
Fatigue fissure and tooth fracture: (**a**,**d**) = fissures in enamel of buccal face of the tooth; (**b**) = a case with fractures of incisal margin of the tooth; (**c**) = a case with vertical crown-root fracture in 4.6.

The diagnosis of dental fractures and the number of teeth with dental fractures (fractured cusps, fractured crowns, or vertical crown–root fractures) was established after dental history and clinical examination using criteria of the American Association of Endodontics [43,52,53]. Clinical aspects of fatigue fractures are presented in Figure 3.

**Figure 4:**
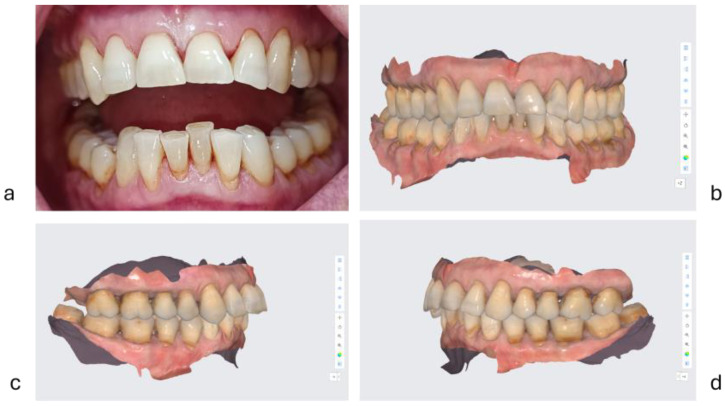
Attrition tooth wear and abfraction lesions in a woman aged 65 years old: (**a**) = clinical image of abfraction lesions on buccal cervical aspects of the teeth; (**b**) = a scanned image with dental arches in occlusion; (**c**) = a scanned image—the right half dental arches; d = a scanned image—the left half dental arches.

**Figure 5:**
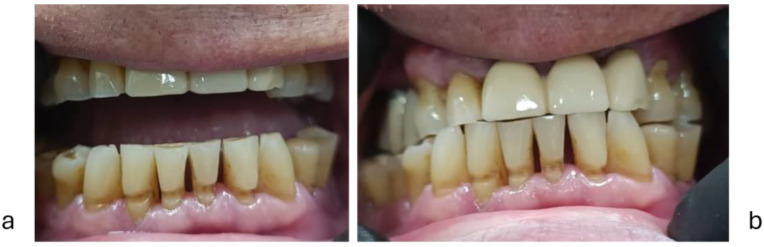
Attrition tooth wear and abfraction lesions in a man aged 64: (**a**,**b**) clinical images of abfraction lesions on buccal cervical aspects of the lower teeth and attrition type tooth wear.

The diagnostic of abfraction was established according to criteria established by the AAE [52], as a V-shaped loss of hard tooth structure caused by biomechanical loading forces [54]. Clinical aspects of abfractions are presented in Figure 4 and Figure 5.

The data obtained about edentulism were classified according to the Eichner index: The Eichner classification (classes A, B, and C) is based on the occlusal contact areas in the antagonist jaws for the natural dentition, including fixed prosthetic restorations. Class A contains four support areas, meaning that there is at least one tooth in contact between the maxilla and mandible in both the premolar and molar regions on each side. Class B contains three (B1), two (B2), or one (B3) support area or the support only in the anterior area (B4). In class C, there are no antagonistic contacts in the dentition. A1—complete dentition; A2—loss of one occlusal unit; A3—loss of two occlusal units on the same arch; B1—loss of one occlusal unit in the terminal edentulous; B2—loss of two occlusal units in the terminal edentulous on the same arch; B3—loss of three occlusal units [55].

**Figure 6:**
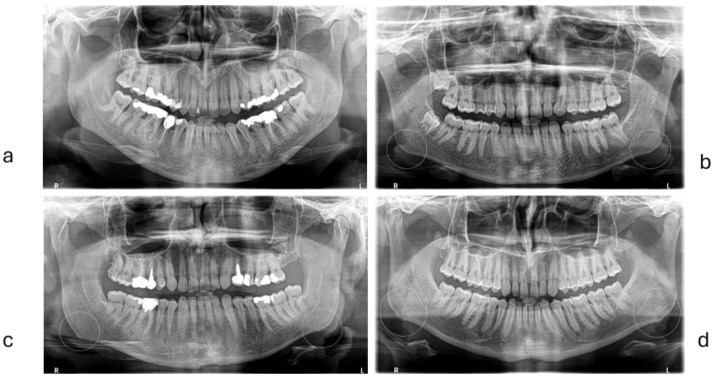
Bone apposition score at mandibular angle: (**a**) = score 0 or absent, (**b**) = score 1 or minor, (**c**) = score 2 or slight, (**d**) = score 3 or major.

Digital panoramic radiographs (orthopantomography) taken with Carestream CS 8100 3D (Carestream Dental LLC, Atlanta, GA, USA) were used to assess bone apposition at the mandible. Bone apposition was identified as a unilateral or bilateral change in the shape of the basal cortical bone at the mandibular angle and classified into four grades (0 = absent, 1 = minor, 2 = slight, and 3 = major) [47,48,56,57], as in Figure 6. The bone apposition score was computed as follows: without bone apposition (patients with scores 0 and/or 1 in both sides), moderate bone apposition (for patients with one angle without bone apposition, one angle with bone apposition 2 or 3), and high bone apposition (for patients with scores of 2 or 3 in both mandibular angles).

### 2.4. Statistical Analysis

The data collected within this study were first centralized in Microsoft Excel (Microsoft Corporation, Redmond, WA, USA). Descriptive statistics were presented as the couple “mean ± standard deviation (SD)” or median values for continuous variables, as well as frequency distributions and associated percentages for nominal and ordinal parameters. Statistical tests were applied with SPSS (Statistical Package for Social Sciences) software, version 26 (SPSS Inc., Armonk, NY, USA). All continuous data series were analyzed for normality based on the Kolmogorov–Smirnov/Shapiro–Wilk test. Based on these results, comparisons between groups for all continuous variables were performed using the Mann–Whitney U and Kruskal–Wallis H tests. Associations were tested using a chi-square test. A binomial regression model was developed to determine the effects of the studied parameters on the probability of patients having bruxism. This model included two demographic variables (age and gender) and seven clinical variables (presence of hypersensitivity, presence of masseter muscle hypertrophy, moderate and high bone apposition scores, the total number of present teeth, the tooth wear index, the number of teeth with abfraction, and the number of fractured teeth). The linearity of all continuous variables with respect to the logit of the dependent variable (bruxism presence) was assessed via the Box–Tidwell (1962) procedure. A Bonferroni correction was applied using all 10 terms in the model, resulting in statistical significance being accepted when *p* < 0.005. Based on this assessment, all continuous independent variables from the model were linearly related to the logit of the dependent variable. All other *p*-values smaller than 0.05 indicated statistically significant results.

## 3. Results

This study included 181 patients, 99 women, and 82 men aged between 20 and 72 years, with a mean age of 44.87 ± 12.67 and a median of 45.00 (Table 1). Group distribution by gender had similarity for variables like number of teeth, tooth wear index, number of teeth with abfractions, number of teeth with fractures, and hypersensitivity, according to Table 1. Significantly more males were smokers (*p* = 0.017), and more males than women had hypertrophy of masseter muscle (*p* = 0.001). High bone apposition scores (similar 2,3) were more frequent for men than for women (*p* < 0.0005), while low bone apposition scores (similar 0,1) were more frequent for women than for men (*p* = 0.001). The Eichner score was significantly different between women and men, for women the decreasing frequency of scores was A3, A1, A2, B2, B1, while for men the decreasing frequency was A1, A3, A2, B2, B1 (*p* < 0.0005), meaning that women had with 6% fewer A1 situations (intact dentition) than men (Table 1).

To appreciate the differences between groups influenced by age, a distribution of all parameters by age groups was computed, and the results are presented in Table 2. The age groups by decade (each ten years from 20 to 70) were analyzed for all other variables except bruxism. Gender and smoking were not influenced by age. Also, the hypersensitivity and bone apposition scores were not significantly different between age groups. The median number of teeth was statistically significantly different between age groups, decreasing constantly from 31 to 25 (*p* < 0.0005). The tooth wear index (TWI), number of teeth with abfraction lesions, and number of teeth with fractures were statistically significantly different between age groups, increasing constantly (*p* < 0.0005 for each of the mentioned variable). The median TWI increased from 1 to 2 (for people over 50 years old). The median number of teeth with abfractions increased from 0 (age group 20–29) to 4 (age group over 60). The median number of teeth with fractures increased from 0 (decade 20) to 1 (decade 30) to 2 (40 and older) (Table 2). Edentulism score was correlated with age groups, increasing with age (*p* < 0.0005). Masseter muscle hypertrophy frequency increased with age, from 30.43% in the decade 20 to 66.67% in decades 40 and over 60, statistically significant (*p* = 0.016). Higher scores of bone apposition (2,3) increased with age, and lower scores of bone apposition (0,1) decreased with age (Table 2).

Depending on the diagnosis of bruxism, the group was divided into patients with bruxism and patients without bruxism, as follows: the group with bruxism included 72 patients (35 women and 37 men) with a mean age of 42.51 ± 13.70 years old, and the group without bruxism included 109 patients (64 women and 45 men) with a mean age of 46.42 ± 11.74 years old. There were no significant differences between the groups in terms of gender and age, as well as smoking (Table 3).

Almost 30% of patients belonged to the age group 40–49 (29.83%), followed by age groups 30–39 (21.55%) and 50–59 (20.99%), and in the end, age groups over 60 (14.92%) and 20–29 years (12.71%). A similar distribution of groups according to age was observed for bruxism patients and no bruxism patients, but the difference between subgroups was higher for bruxism compared with no bruxism group (Table 3).

**Figure 7:**
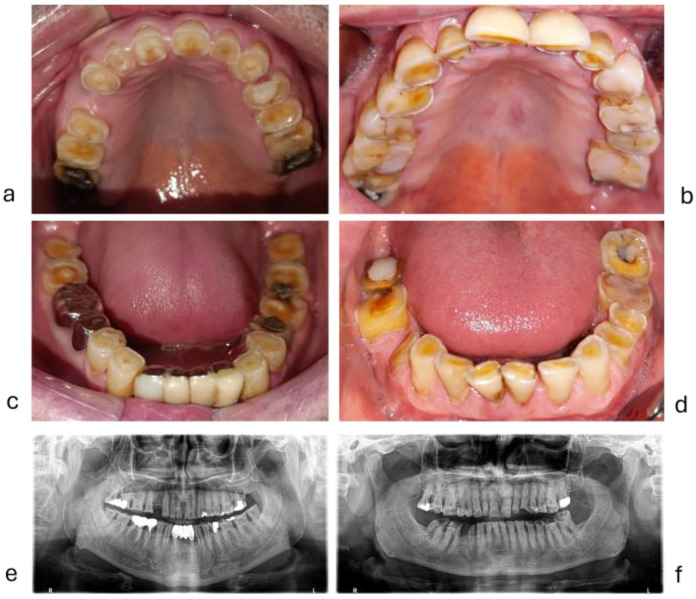
Extreme tooth wear in two men: (**a**,**c**,**e**) = a case with extreme tooth wear in centric bruxism; (**b**,**d**,**f**) = a case with extreme tooth wear in excentric bruxism.

The two groups of patients, with and without bruxism, presented similarities in the number of teeth present, the difference between the two groups being statistically insignificant. The number of teeth for all patients was between 18 and 32, with a mean of 27.29 ± 3.47 and a median of 28. The median number of teeth in the bruxism group was 27, while in the group without bruxism, this value was 28. The tooth wear index in the studied groups was between 1 and 3, with a mean index of 1.76 ± 0.47. Regarding the tooth wear, a statistically significant difference was recorded between the two groups, the tooth wear index being significantly higher in the group with bruxism (median 2) than in the group without bruxism (median 1.6), *p* < 0.0005 (Table 4). A part of the group had extreme tooth wear (Figure 7). The number of teeth with abfraction lesions varied between 0 and 9 with a mean of 2 ± 1.94. The number of teeth with abfraction lesions was higher in the group with bruxism (median 2.5) compared to the group without bruxism (median 2), *p* = 0.001 (Table 4). The number of teeth with fractures of cusps, crown, or vertical tooth fracture varied between 0 and 6 with a mean of 1.59 ± 1.26. The number of teeth with dental fractures was significantly higher in the group with bruxism (median 2) compared to the group without bruxism (median 1), *p* = 0.037 (Table 4).

The Eichner edentulous score was statistically significantly different between the two groups, the types of edentulousness being found in both groups in different percentages, and different frequency order: in the bruxism group, the decreasing frequency was A1, A3, A2, B1, B2, while for the no bruxism group, the decreasing frequency was A3, A1, A2, B2, B1 (*p* < 0.0005). This frequency showed that bruxism people had more occlusal contacts than no bruxism people, even though the median number of teeth was 27 in the bruxism group and 28 in the no bruxism group (Table 4).

Regarding the presence of dental hypersensitivity, there were no statistically significant differences between the two groups, with the percentages of patients who had hypersensitivity being relatively similar between the two groups, *p* = 0.409 (Table 4).

Masseter muscle hypertrophy was found much more frequently in patients with bruxism (91.67% of them presenting masseter hypertrophy) compared to the group without bruxism, who had hypertrophy in a percentage of 31.19%, *p* < 0.0005 (Table 4).

Overall bone apposition scores were opposed distributed between patients with and without bruxism (Table 4). Bone apposition was encountered in 89.50% of the entire studied group, and the number of patients with a score of 0 and 1 (changes in bone not important) was 72 (39.77%), more in the group without bruxism. Regarding bone apposition at the mandibular angle, there were statistically significant differences between the groups, as follows: the group of patients with bruxism presented more statistically significant scores of 2 and 3 of bone apposition (86.11%), (*p* < 0.0005) compared to the group of patients without bruxism, who presented a high bone apposition score in the percentage of 28.44% (Table 4). Bone apposition was usually present bilaterally in low scores, or in high scores, in the right angle or left angle, or both, with statistically significant differences between the two groups (*p* < 0.0005) (Table 4).

In Table 5, all variables analyzed by bruxism and gender are presented. Median age, age groups, number of present teeth, Eichner score, and hypersensitivity did not differ between women with bruxism and women without bruxism. Masseter muscle hypertrophy, tooth wear index, number of teeth with abfraction, and bone apposition score were statistically significantly different between the two groups of women, with and without bruxism, their values being higher for women with bruxism. Also, number of teeth with fractures were twice more frequent in bruxism women compared to no bruxism women (Table 5).

For men, median age, age groups, Eichner score, hypersensitivity, and number of teeth with fractures were not significantly different between men with bruxism and men without bruxism. Similarly to women, the masseter muscle hypertrophy, tooth wear index, number of teeth with abfraction, and bone apposition scores were statistically significantly different between the two groups of men, with and without bruxism, their values being high for men with bruxism. The number of present teeth was higher for men without bruxism (*p* = 0.043). The number of teeth with fractures was similar in bruxism men compared to no bruxism men (Table 5).

Table 6 presents the characteristics of the two groups (with/without bruxism) according to age groups. For the first age group (20–29 years), most variables were similar between the two groups, except masseter muscle hypertrophy and bone apposition scores, which were significantly high in the bruxism group. Masseter muscle hypertrophy was statistically significantly different between bruxism and no bruxism groups for all age groups, having a higher frequency in bruxism (*p* < 0.0005). The bone apposition score was higher in patients with bruxism in all age groups, compared to patients without bruxism, that mainly had low bone apposition scores. The moderate bone apposition score (with one angle low and one angle high) was more frequent in no bruxism patients. The tooth wear index was significantly statistically higher in the bruxism group than in the no bruxism group for all age groups from 30 years old, as follows: *p* = 0.009 for decade 30–39 years old, *p* = 0.044 for decade 40–49 years old, *p* = 0.006 for decade 50–59 years old, and *p* = 0.005 for patients with ages > 60 years old (Table 6). The number of teeth with abfraction was higher in age groups in decades 30–39, 40–49, and over 60.

The association between bruxism, masseter hypertrophy, bone apposition in mandibular angle, extreme attrition tooth wear, abfractions, fatigue fissure, and tooth fracture are presented in two cases in Figure 7.

### Bruxism Prediction

A binomial logistic regression was performed to ascertain the effects of gender, age, total number of present teeth, tooth wear index, number of fractured teeth, number of teeth with abfraction, presence of hypersensitivity, presence of masseter hypertrophy, and bone apposition scores of 2 or 3 on at least one side, on the likelihood that patients having bruxism. There were no standardized residuals with value less than ±2.5. The logistic regression model was statistically significant, χ^2^(9) = 93.196, *p* < 0.0005. The model explained 54.40% (Nagelkerke R2) of the variance in bruxism and correctly classified 80.70% of cases. The sensitivity was 80.55%, the specificity was 80.73%, the positive predictive value was 73.41%, and the negative predictive value was 86.27%. Of the ten predictor variables, only five were statistically significant: masseter muscle hypertrophy, bone apposition score, age, tooth wear index, and the number of teeth with abfraction (Table 7).

Patients with masseter muscle hypertrophy had 15-times-higher odds of exhibiting bruxism than patients without masseter hypertrophy. Patients with a moderate or high bone apposition score had almost 3-times-higher odds of exhibiting bruxism than patients without bone apposition. Increasing the tooth wear index was associated with an increased likelihood of exhibiting bruxism (for an increase in the tooth wear index by one unit, the odds of having bruxism increase by a factor of 6.411); similarly, an increasing number of teeth with abfraction was associated with an increased probability of exhibiting bruxism (by a factor of 1.286), but increasing age was associated with a minimal reduction in the probability of exhibiting bruxism.

## 4. Discussion

The objective of the cross-sectional clinical study was to highlight the association of bruxism with several oral clinical signs correlated with excessive occlusal forces (masseter hypertrophy, attrition-type tooth wear, tooth abfraction, dental fatigue fractures) and radiological (bone apposition at the level of the mandibular angles). The subject of the present study is of interest to several medical fields that treat bruxism. In the revised literature, most publications treat especially the aspects of bruxism correlated with temporomandibular dysfunction [58,59], orofacial pain [60,61], or sleep disorders [62,63], while the oral consequences of bruxism and excessive forces are less studied. The present study comes with a novelty in the field, namely the role of OPG radiological evaluation in the diagnosis of bruxism, a diagnostic element not found in the latest STAB document developed with the aim of establishing the diagnostic criteria for bruxism [49]. Radiological signs are analyzed in correlation with oral and dental signs of the consequences of excessive occlusal forces from bruxism, from masseter hypertrophy to dental attrition, dental abfraction, and dental fatigue fractures.

The binomial logistic regression model results from the current study showed that the presence of masseter muscle hypertrophy increased the odds of exhibiting bruxism 15 times (*p* < 0.0005, CI [4.457, 53.765]), and the presence of a higher tooth wear index increased the odds of having bruxism approximately 7 times (*p* = 0.010, CI [1.545, 26.609]). Clinical examination plays a very important role in bruxism diagnostic, especially in clinical setting, when long questionnaires are not so helpful. The correlation of these two factors, masseter muscle hypertrophy and attrition tooth wear with bruxism, showed that mechanical factors are important in tooth wear etiopathogenesis. Excessive occlusal forces in bruxism correlate with a higher tooth wear index and a higher masseter muscle force exercised by a higher muscle volume. The masseter muscle hypertrophy was also correlated with a high bone apposition score at the mandibular angle. The bone apposition in the mandibular angle could be considered a radiological sign of bruxism, which is easier to identify on digital orthopantomography (a very often used investigation in general dentistry) [47,48]. A high bone apposition score increases 3 times the odds of patients having bruxism. These correlations are present in all adult age groups (20 to 70 years old). This model was supported by the results of the present clinical study that showed that in patients with bruxism, the tooth wear index was statistically significantly higher than in patients without bruxism. Other clinical signs of excessive occlusal forces, like the number of fractured teeth and the number of teeth with abfraction lesions, were also statistically significantly higher in patients with bruxism compared to patients without bruxism.

The current study showed a powerful association between masseter muscle hypertrophy and bruxism in both men and women. Similar results were obtained by other studies, like the one recently published by Scheibel et al. in 2025 [64]. In a population-based magnetic resonance imaging study on 720 subjects aged 30–89 years old from Pomerania, North Germany, linear and ordinal logistic regression models showed that a larger masseter cross-sectional area (4.68 ± 0.98 cm^2^ in men; 3.56 ± 0.77 cm^2^ in women) was significantly associated with bruxism in men64. In our study, bruxism frequency was also influenced by gender, but in a small manner; although women had bruxism in a smaller percentage (37%) than men (43%), the difference was insignificant. As for masseter hypertrophy, both women and men had a significant association of masseter muscle hypertrophy with bruxism (*p* < 0.0005). This finding correlated with a significantly higher clenching activity in bruxism, observed in other studies performed by our group on a population of the same origin (from Oltenia, South Romania) [5,7]. The electromyographic activity recorded by surface EMG on 24 h showed a higher masseter muscle activity in people with bruxism of any type, awake or sleep [5,65,66].

A higher bone apposition score was statistically significantly frequent in patients with bruxism than those without bruxism. Radiological aspects of bruxism that appear as bone apposition in mandibular angles because of increased masseter muscle forces represent radiological aspects of bruxism and excessive muscular masseteric activity, as some recent research pointed out [47,48]. The number of patients with masseter muscle hypertrophy was also statistically significantly higher in the bruxism group compared to the no bruxism group, and that was correlated with the bone apposition score. Clinical signs present in patients with bruxism showed that masseter muscle hypertrophy was positively correlated with a high bone apposition score and with tooth wear index and the number of teeth with abfraction, which means that excessive occlusal forces (as intensity and/or duration) have long-term adverse effects on hard dental structures (enamel, dentin).

Regarding the correlation of the bone apposition score with age, it was observed that in all age groups, the bone apposition score was high in the bruxism group, being statistically significantly more frequent in bruxers between 20 and 49 years old and from 60 years old. In the 50–59-year-old group, the number of people without bruxism was 3 times higher than those with bruxism, and this is probably the reason for the lack of statistical significance.

Occlusal-attrition-type and cervical-abfraction-type tooth wear were correlated with occlusal stress and bruxism [54,67,68]. In a population-based cohort study called The Study of Health in Pomerania (SHIP-START), in which attrition and abfraction and their cross-sectional and longitudinal association were evaluated over a 16-year period, it was observed that the presence of occlusal wear increased the probability of a large non-carious cervical lesion of the abfraction type by 7 times [69]. In a stereomicroscopy study performed on teeth extracted with abfraction, the presence of cracks in the cervical lesion was observed, which denotes the role of occlusal overloads in the etiology of abfraction [70]. The association between bruxism and excessive occlusal forces was observed in subjects with bruxism diagnosed by sEMG [7].

In subjects with sleep bruxism and reporting sounds during sleep, the presence of marked attrition was accompanied by a much higher number of phasic contraction episodes accompanied by sound production compared to subjects with sleep bruxism in whom no sound production was recorded. In addition to the high number, the phasic contraction episodes also had longer durations [33]. In 2014, Karakis et al. [71] measured bite force. Their study showed that this force was not different in women with bruxism compared to those without bruxism, while in men with bruxism, the force was significantly higher than in men without bruxism. They observed a negative correlation between CMI (craniomandibular index) and bite force in subjects of both sexes. Women with bruxism had a higher CMI than men with bruxism, which results in lower bite forces.

In the current study, the number of teeth with abfraction was significantly higher in patients with bruxism compared to those without bruxism. Other studies have also observed the same thing, with the note that, in some patients with sleep bruxism, the number of teeth affected by abfraction lesions was much higher [72,73]. Dental stress fractures have bruxism as their etiology [26,74]. The number of fractured teeth increases with age due to the phenomenon of tooth aging [44,75].

Is worth mentioning that, in the comparison between men and women, men had a significantly higher frequency of masseter hypertrophy. This finding could be explained by a higher masseter muscle thickness in men compared with women [64]. Also, the individuals with hypodivergent facial morphology had a masseter muscle thicker than other groups [76]. In our study, the frequency of smoking in men was higher than in women, and this could be correlated with a different response to a higher level of stress [77].

Regarding age groups, if the gender distribution in these age groups was similar, and smoking was not a factor, all other variables showed statistically significant differences between age groups. Thus, masseter hypertrophy and bone apposition score increased with age in frequency, as did the tooth wear index, the number of fractured teeth, and the number of teeth with abfraction, as well as the edentulous score. Aging accentuates the effects of excessive occlusal forces from bruxism, and as the years pass, more and more teeth are lost due to their destruction by wear and fractures.

Masseter hypertrophy and bone apposition at the mandibular angles, correlated with attrition, abfraction, and dental fractures, are associated with the fact that in bruxism, the intensity of clenching activity increases [5,7], a specific activity of the masseter muscles that increases muscle volume [78].

The study is unique in the literature because it associates clinical criteria with anamnestic and radiological ones for the diagnosis of bruxism. Compared to the latest proposal of the international bruxism research group, STAB 2024 [49], the use of these clinical and radiological criteria for diagnosing bruxism represents a simple and objective diagnostic scheme. Thus, the association of masseter hypertrophy with mandibular bone apposition of score 2 or/and 3, with attrition-type dental wear over 2 and with dental abfractions in over two teeth, as well as with dental fatigue fractures, to which are added anamnestic criteria (noises produced during sleep or awareness of teeth clenching during the day), can represent clear diagnostic elements for bruxism. It should be noted that, in the case of bruxist patients who present to the dental office for dental treatment, most of them do not know how to declare the existence of bruxism. After the dentist asks them questions regarding bruxism and makes them aware of its existence, the patients become aware of the presence of bruxism, which they recognize. A general dentist who sees an OPG with changes in the mandibular angles and the clinical signs characteristic of bruxism (masseter hypertrophy, attrition, abfraction, fractured teeth) can much more easily diagnose bruxism.

### Study Limitations

- No differentiation was made between sleep and awake bruxism in the patients in this study. The data processed were extracted from the dental chart, not from specific bruxism questionnaires.
- Bruxism was not diagnosed by instrumental criteria of video-polysomnography or electromyography, because in the general dentistry office level, it is very difficult to do this.
- No long-term evaluation of the subjects included in the study was made, as the study was cross-sectional and monocentric.

## 5. Conclusions

Our study showed that patients with masseter muscle hypertrophy had 15-times-higher odds of exhibiting bruxism than patients without masseter hypertrophy. Patients with a moderate or high bone apposition score had almost 3-times-higher odds of exhibiting bruxism than patients without bone apposition. Increasing tooth wear index and number of teeth with abfraction were associated with an increased probability of exhibiting bruxism.

Excessive forces in bruxism have clinical consequences on hard dental structures (enamel and dentine), causing teeth destruction. These oral effects were sustained by the association of bruxism with tooth wear (attrition), tooth fractures, and abfractions, as well as with masseter hypertrophy and bone apposition in mandibular angles. Bruxism is strongly predicted by masseter muscle hypertrophy, high bone apposition score, and high tooth wear index. Such simple and reliable clinical and radiological criteria for bruxism diagnostic are necessary in dental office settings. With advancing age, the clinical signs of bruxism become more evident as a consequence of the cumulative effects of excessive forces. All dentists (of any specialty) should observe and diagnose bruxism, to try to manage and prevent this type of tooth destruction, using occlusal appliances and other means of bruxism management.

## Figures and Tables

**Table 1:** Distribution of the study parameters, divided by gender.

Parameter	Values	Females	Males	Total	*p*
		99 Patients	82 Patients	181 Patients	
Age groups (years old)	20–29	10 (43.48%)	13 (56.52%)	23 (100%)	0.116 *
		10.10%	15.85%		
	30–39	23 (58.97%)	16 (41.03%)	39 (100%)	
		23.23%	19.51%		
	40–49	31 (57.41%)	23 (42.59%)	54 (100%)	
		31.31%	28.05%		
	50–59	21 (55.26%)	17 (44.74%)	38 (100%)	
		21.21%	20.73%		
	≥60	14 (51.85%)	13 (48.15%)	27 (100%)	
		14.14%	15.85%		
Smoking	Yes	30 (43.48%)	39 (56.52%)	69 (100%)	0.017 *
		30.30%	47.56%		
	No	69 (61.61%)	43 (38.39%)	112 (100%)	
		69.70%	52.44%		
Number of present teeth	Median	27.00	28.00	-	0.228 **
Tooth wear index	Median	1.70	1.80	-	0.142 **
Number of teeth with abfraction	Median	2.00	2.00	-	0.240 **
Number of teeth with fractures	Median	1.00	2.00	-	0.578 **
Eichner score	A1	27 (50.00%)	27 (50.00%)	54 (100%)	<0.0005 *
		27.27%	32.93%		
	A2	25 (59.52%)	17 (40.48%)	42 (100%)	
		25.25%	20.73%		
	A3	32 (56.14%)	25 (43.86%)	57 (100%)	
		32.32%	30.49%		
	B1	4 (40.00%)	6 (60.00%)	10 (100%)	
		4.04%	7.32%		
	B2	11 (61.11%)	7 (38.89%)	18 (100%)	
		11.11%	8.54%		
Hypersensitivity	Yes	27 (64.29%)	15 (35.71%)	42 (100%)	0.154 *
		27.27%	18.29%		
	No	72 (51.80%)	67 (48.20%)	139 (100%)	
		72.73%	81.71%		
Masseter muscle hypertrophy	Yes	44 (44%)	56 (56%)	100 (100%)	0.001 *
		44.44%	68.29%		
	No	55 (67.90%)	26 (32.10%)	81 (100%)	
		55.56%	31.71%		
Bone apposition score	Without	43 (59.72%)	29 (40.28%)	72 (100%)	0.340 *
		43.43%	35.37%		
	Moderate	10 (62.50%)	6 (37.50%)	16 (100%)	
		10.10%	7.32%		
	High	46 (49.46%)	47 (50.54%)	93 (100%)	
		46.46%	57.32%		

* Chi-square test. ** Mann–Whitney U test. Values emphasized in light grey represent the sum of values by column.

**Table 2:** Distribution of the study parameters, divided by age groups (expressed in years old).

Parameter	Values	20–29	30–39	40–49	50–59	≥60	Total	*p*
		23 Patients	39 Patients	54 Patients	38 Patients	27 Patients	181 Patients	
Gender	Females	10 (10.10%)	23 (23.23%)	31 (31.31%)	21 (21.21%)	14 (14.14%)	99 (100%)	0.789 *
		43.48%	58.97%	57.41%	55.26%	51.85%		
	Males	13 (15.85%)	16 (19.51%)	23 (28.05%)	17 (20.73%)	13 (15.85%)	82 (100%)	
		56.52%	41.03%	42.59%	44.74%	48.15%		
Smoking	Yes	9 (13.04%)	17 (24.64%)	24 (34.78%)	11 (15.94%)	8 (11.59%)	69 (100%)	0.463 *
		39.13%	43.59%	44.44%	28.95%	29.63%		
	No	14 (12.50%)	22 (19.64%)	30 (26.79%)	27 (24.11%)	19 (16.96%)	112 (100%)	
		60.87%	56.41%	55.56%	71.05%	70.37%		
Number of present teeth	Median	31.00	29.00	28.00	25.00	25.00	-	<0.0005 **
Tooth Wear Index	Median	1.00	1.40	1.80	2.00	2.00	-	<0.0005 **
Number of teeth with abfraction	Median	0.00	1.00	2.00	2.00	4.00	-	<0.0005 **
Number of teeth with fractures	Median	1.00	1.00	2.00	2.00	2.00	-	<0.0005 **
Eichner score	A1	9 (16.67%)	11 (20.37%)	18 (33.33%)	12 (22.22%)	4 (7.41%)	54 (100%)	<0.0005 *
		39.13%	28.21%	33.33%	31.58%	14.81%		
	A2	6 (14.29%)	5 (11.90%)	18 (42.86%)	6 (14.29%)	7 (16.67%)	42 (100%)	
		26.09%	12.82%	33.33%	15.79%	25.93%		
	A3	5 (8.77%)	13 (22.81%)	13 (22.81%)	15 (26.32%)	11 (19.30%)	57 (100%)	
		21.74%	33.33%	24.07%	39.47%	40.74%		
	B1	1 (10.00%)	3 (30.00%)	1 (10.00%)	1 (10.00%)	4 (40.00%)	10 (100%)	
		4.35%	7.69%	1.85%	2.63%	14.81%		
	B2	2 (11.11%)	7 (38.89%)	4 (22.22%)	4 (22.22%)	1 (5.56%)	18 (100%)	
		8.70%	17.95%	7.41%	10.53%	3.70%		
Hypersensitivity	Yes	7 (16.67%)	11 (26.19%)	13 (30.95%)	5 (11.90%)	6 (14.29%)	42 (100%)	0.491 *
		30.43%	28.21%	24.07%	13.16%	22.22%		
	No	16 (11.51%)	28 (20.14%)	41 (29.50%)	33 (23.74%)	21 (15.11%)	139 (100%)	
		69.57%	71.79%	75.93%	86.84%	77.78%		
Masseter muscle hypertrophy	Yes	7 (7.00%)	17 (17.00%)	36 (36.00%)	22 (22.00%)	18 (18.00%)	100 (100%)	0.016 *
		30.43%	43.59%	66.67%	57.89%	66.67%		
	No	16 (19.75%)	22 (27.16%)	18 (22.22%)	16 (19.75%)	9 (11.11%)	81 (100%)	
		69.57%	56.41%	33.33%	42.11%	33.33%		
Bone apposition score	Without	4 (5.56%)	19 (26.39%)	15 (20.83%)	18 (25.00%)	16 (22.22%)	72 (100%)	0.030 *
		17.39%	48.72%	27.78%	47.37%	59.26%		
	Moderate	3 (18.75%)	3 (18.75%)	4 (25.00%)	5 (31.25%)	1 (6.25%)	16 (100%)	
		13.04%	7.69%	7.41%	13.16%	3.70%		
	High	16 (17.20%)	17 (18.28%)	35 (37.63%)	15 (16.13%)	10 (10.75%)	93 (100%)	
		69.57%	43.59%	64.81%	39.47%	37.04%		

* Chi-square test. ** Mann–Whitney U test. Values emphasized in light grey represent the sum of values by column.

**Table 3:** Demographic characteristics of the two study groups: bruxism vs. no bruxism.

Parameter	Values	Bruxism	No Bruxism	Total	*p* *
		72 Patients	109 Patients	181 Patients	
Gender	F	35 (35.35%)	64 (64.65%)	99 (100%)	0.181
		48.61%	58.72%		
	M	37 (45.12%)	45 (54.88%)	82 (100%)	
		51.39%	41.28%		
Age group (years old)	20–29	16 (69.57%)	7 (30.43%)	23 (100%)	0.055
		22.22%	6.42%		
	30–39	14 (35.90%)	25 (64.10%)	39 (100%)	
		19.44%	22.94%		
	40–49	22 (40.74%)	32 (59.26%)	54 (100%)	
		30.56%	29.36%		
	50–59	10 (26.32%)	28 (73.68%)	38 (100%)	
		13.89%	25.69%		
	≥60	10 (37.04%)	17 (62.96%)	27 (100%)	
		13.89%	15.60%		
Smoking	Yes	25 (36.23%)	44 (63.77%)	69 (100%)	0.483
		34.72%	40.37%		
	No	47 (41.96%)	65 (58.04%)	112 (100%)	
		65.28%	59.63%		

* Chi-square test. Values emphasized in light grey represent the sum of values by column.

**Table 4:** Characteristics of the study groups as resulted from clinical exam.

Parameter	Values	Bruxism	No Bruxism	Total	*p*
		72 Patients	109 Patients	181 Patients	
Number of present teeth	Median	27.00	28.00	-	0.425 *
Tooth Wear Index	Median	2.00	1.60	-	<0.0005 *
Number of teeth with abfraction	Median	2.50	2.00	-	0.001 *
Number of teeth with fractures	Median	2.00	1.00	-	0.037 *
Eichner score	A1	19 (35.19%)	35 (64.81%)	54 (100%)	
		26.39%	32.11%		
	A2	18 (42.86%)	24 (57.14%)	42 (100%)	
		25.00%	22.02%		
	A3	24 (42.11%)	33 (57.89%)	57 (100%)	0.942 **
		33.33%	30.28%		
	B1	4 (40.00%)	6 (60.00%)	10 (100%)	
		5.56%	5.50%		
	B2	7 (38.89%)	11 (61.11%)	18 (100%)	
		9.72%	10.09%		
Hypersensitivity	Yes	16 (38.10%)	26 (61.90%)	42 (100%)	0.799 **
		22.22%	23.85%		
	No	56 (40.29%)	83 (59.71%)	139 (100%)	
		77.78%	76.15%		
Masseter muscle hypertrophy	Yes	66 (66.00%)	34 (34.00%)	100 (100%)	<0.0005 **
		91.67%	31.19%		
	No	6 (7.41%)	75 (92.59%)	81 (100%)	
		8.33%	68.81%		
Bone apposition score	Without	6 (8.33%)	66 (91.67%)	72 (100%)	<0.0005 **
		8.33%	60.55%		
	Moderate	4 (25.00%)	12 (75.00%)	16 (100%)	
		5.56%	11.01%		
	High	62 (66.67%)	31 (33.33%)	93 (100%)	
		86.11%	28.44%		

* Mann–Whitney U test. ** Chi-square test. Values emphasized in light grey represent the sum of values by column.

**Table 5:** Characteristics of the study groups, divided by gender.

Parameter	Values		Bruxism	No Bruxism	Total	*p*		Bruxism	No Bruxism	Total	*p*
			35 Females	64 Females	99 Females			37 Males	45 Males	82 Males	
Age group	20–29	**FEMALES**	7	3	10		**MALES**	9	4	13	
	30–39		7	16	23	0.088 *		7	9	16	0.408 *
	40–49		12	19	31			10	13	23	
	50–59		4	17	21			6	11	17	
	>60		5	9	14			5	8	13	
Eichner score	A1		7	20	27			12	15	27	
	A2		10	15	25	0.705 *		8	9	17	0.805 *
	A3		11	21	32			13	12	25	
	B1		2	2	4			2	4	6	
	B2		5	6	11			2	5	7	
Hypersensitivity	Yes		10	17	27	0.830 *		6	9	15	0.659 *
	No		25	47	72			31	36	67	
Masseter muscle hypertrophy	Yes		33	20	53	<0.0005 *		33	14	47	<0.0005 *
	No		2	44	46			4	31	35	
Bone apposition score	Without		4	39	43	<0.0005 *		2	27	29	
	Moderate		3	7	10			1	5	6	<0.0005 *
	High		28	18	46			34	13	47	
Age	median		46.00	45.50	-	0.854 **		48.00	44.00	-	0.105 **
Number of present teeth	median		28.00	27.00	-	0.497 **		27.00	30.00	-	0.043 **
Tooth wear index	median		1.80	1.60	-	0.010 **		2.00	1.60	-	<0.0005 **
Number of teeth with abfraction	median		3.00	2.00	-	0.003 **		2.00	1.00	-	0.042 **
Number of teeth with fractures	median		2.00	1.00	-	0.074 **		2.00	2.00	-	0.260 **

* Chi-square test. ** Mann–Whitney U test.

**Table 6:** Characteristics of the study groups, divided by age group.

Parameter	Values		B	NoB	Total	*p*		B	NoB	Total	*p*		B	NoB	Total	*p*		B	NoB	Total	*p*		B	NoB	Total	*p*
			16 pt	7 pt	23 pt			14 pt	25 pt	39 pt			22 pt	32 pt	54 pt			10 pt	28 pt	38 pt			10 pt	17 pt	27 pt	
Gender	F	**20–29 years old**	7	3	10	0.968 *	**30–39 years old**	7	16	23	0.394 *	**40–49 years old**	12	19	31	0.724 *	**50–59 years old**	4	17	21	0.258 *	**≥60 years old**	5	9	14	0.883 *
	M		9	4	13			7	9	16			10	13	23			6	11	17			5	8	13	
Eichner score	A1		6	3	9			3	8	11			5	13	18			4	8	12			1	3	4	
	A2		4	2	6	0.892 *		1	4	5	0.799 *		9	9	18	0.475 *		1	5	6	0.917 *		3	4	7	0.823 *
	A3		4	1	5			6	7	13			5	8	13			4	11	15			5	6	11	
	B1		1	0	1			1	2	3			1	0	1			0	1	1			1	3	4	
	B2		1	1	2			3	4	7			2	2	4			1	3	4			0	1	1	
Hypersensitivity	Yes		5	2	7	0.898 *		2	9	11	0.266 *		5	8	13	0.848 *		2	3	5	0.456 *		2	4	6	0.831 *
	No		11	5	16			12	16	28			17	24	41			8	25	33			8	13	21	
Masseter muscle hypertrophy	Yes		16	1	17	<0.0005 *		14	4	18	<0.0005 *		20	18	38	0.006 *		7	9	16	0.037*		9	2	11	<0.0005 *
	No		0	6	6			0	21	21			2	14	16			3	19	22			1	15	16	
Bone apposition score	Without		1	3	4	0.018 *		0	19	19			2	13	15			2	16	18			1	15	16	
	Moderate		1	2	3			0	3	3	<0.0005 *		1	3	4	0.021 *		1	4	5	0.065 *		1	0	1	<0.0005 *
	High		14	2	16			14	3	17			19	16	35			7	8	15			8	2	10	
Number of present teeth	median		30.00	31.50	-	0.579 **		29.00	29.50	-	0.988 **		27.00	29.00	-	0.305 **		26.00	25.00	-	0.649 **		25.00	25.00	-	0.720 **
Tooth wear index	median		1.40	1.00	-	0.624 **		1.80	1.40	-	0.009 **		1.80	1.60	-	0.044 **		2.00	2.00	-	0.006 **		2.60	1.90	-	0.005 **
Number of teeth with abfraction	median		0.00	0.00	-	0.413 **		2.00	0.00	-	0.021 **		3.00	2.00	-	0.057 **		2.00	2.00	-	0.465 **		4.00	2.00	-	0.076 **
Number of teeth with fractures	median		1.00	1.50	-	0.278 **		2.00	1.00	-	0.142 **		2.00	2.00	-	0.226 **		2.00	2.00	-	0.222 **		1.50	2.00	-	0.488 **

* Chi-square test and ** Mann–Whitney U test for median values. B = bruxism; NoB = No bruxism; pt = patients.

**Table 7:** Logistic regression predicting the likelihood of bruxism based on demographics and clinical data.

Parameter	B ^1^	Sig	Exp(B)	CI Interval for Exp(B)	
				Lower	Upper
Gender	0.785	0.085	2.191	0.897	5.351
Hypersensitivity	0.398	0.425	1.489	0.561	3.953
Masseter muscle hypertrophy	2.740	<0.0005	15.481	4.457	53.765
Bone apposition score	0.978	0.044	2.658	1.029	6.869
Age	−0.068	0.010	0.934	0.887	0.984
Number of present teeth	0.016	0.831	1.016	0.876	1.179
Tooth wear index	1.858	0.010	6.411	1.545	26.609
Number of teeth with abfraction	0.251	0.044	1.286	1.006	1.643
Number of teeth with fractures	−0.256	0.168	0.774	0.538	1.114

^1^ B coefficients predict the probability of occurring bruxism for a patient. Exp(B) indicates the change in the odds of occurring bruxism for each switch of the parameter; for the last five parameters, it represents the change in the odds of occurring bruxism for each increase in one unit. Sig represents the statistical significance of the test.

## Data Availability

The authors declare that the data of this research are available from the corresponding authors upon reasonable request.
